# Genetic analysis of common triazole resistance mechanisms in a collection of *Aspergillus lentulus* clinical isolates from the United States

**DOI:** 10.1128/aac.00690-25

**Published:** 2025-09-12

**Authors:** Adela Martin-Vicente, Ashley V. Nywening, Jinhong Xie, Harrison I. Thorn, Xabier Guruceaga, Jarrod R. Fortwendel

**Affiliations:** 1 Department of Clinical Pharmacy and Translational Science, University of Tennessee Health Science Center 550089, Memphis, Tennessee, USA; 2 Department of Pediatrics, College of Medicine, University of Arkansas for Medical Sciences 12215, Little Rock, Arkansas, USA; 3 Graduate Program in Pharmaceutical Sciences, College of Pharmacy, University of Tennessee Health Science Center 12326, Memphis, Tennessee, USA; 4 Department of Agronomy, Biotechnology and Food, University of Navarra 16754, Pamplona, Spain; 5 Department of Microbiology, Immunology, and Biochemistry, College of Medicine, University of Tennessee Health Science Center 274062, Memphis, Tennessee, USA; University of Iowa, Iowa City, Iowa, USA

**Keywords:** *Aspergillus lentulus*, triazole resistance

## Abstract

*Aspergillus fumigatus* continues to be the leading cause of invasive aspergillosis. However, the number of cases by drug-resistant cryptic species has increased in recent years. *Aspergillus lentulus* is a sibling species of *Aspergillus* section *Fumigati* that can only be distinguished from *A. fumigatus* by molecular methods. The clinical importance of this species resides in its low susceptibility to triazoles and intrinsic resistance to amphotericin B, making invasive aspergillosis treatments extremely challenging and producing high mortality rates. In this study, we investigate known molecular mechanisms important for triazole resistance in *A. fumigatus* in a collection of 25 clinical *A. lentulus* isolates from the United States. Using CRISPR-Cas9 gene editing technology, we performed *cyp51A* and *hmg1* allele replacements between susceptible and resistant isolates. Phenotypic characterization of the resulting mutants, together with mRNA expression analyzes of *cyp51A*, *cyp51B,* and the putative ABC efflux pump, *abcC*, suggests that triazole resistance in our *A. lentulus* isolates is independent of the mechanisms studied.

## INTRODUCTION

Invasive aspergillosis (IA) is the most serious and life-threatening disease caused by members of the genus *Aspergillus* and affects around two million people every year with high medical costs and elevated mortality rates (1). Within this genus, *Aspergillus fumigatus* continues to be the leading species causing disease and has recently been included on the World Health Organization fungal priority pathogens list (2).

Taxonomy of the genus *Aspergillus* is highly complex, but studies have divided this genus into eight subgenera and 25 sections, based on phenotypic characteristics (3–5). *A. fumigatus* belongs to the section *Fumigati*, which contains around 60 species that cannot be differentiated from *A. fumigatus* by traditional morphological analyzes. Although *A. fumigatus sensu stricto* remains the most common cause of IA, cryptic species also cause IA, but their prevalence is not well understood due to diagnostic limitations. Studies from the United States, South Korea, Japan, and Spain found that around 10%–30% of cases of aspergillosis may be caused by cryptic species (6–12). Some of these cryptic aspergilli are intrinsically resistant or present reduced susceptibility to amphotericin B (AMB) and triazoles (6, 13–15), making their treatment extremely challenging if an early identification is not achieved. One of the most commonly identified cryptic species in cases of IA is *Aspergillus lentulus,* which was first reported as a new species in 2005 by Balajee et al. (16). While it presents almost identical macro and micromorphology to *A. fumigatus*, the genetic distance from *A. lentulus* is evident when using a multi-locus sequencing approach (17). The most clinically relevant characteristic of *A. lentulus* is its intrinsic resistance to AMB and its reduced susceptibility to the treatment of choice for IA, voriconazole (VRC), as well as to the other triazoles (6, 14, 18–20). Several case reports have described *A. lentulus* as the causative agent of aspergillosis in both neutropenic and non-neutropenic patients, and the mortality rates associated with this organism are over 50%, despite antifungal treatment (18, 21–31).

In the last few decades, much research has focused on understanding the molecular processes that drive antifungal resistance in *A. fumigatus*. A variety of mutations in the coding region of *cyp51A*, encoding for the target of triazoles, as well as tandem repeat promoter mutations of this gene, represent the most common mechanisms of triazole resistance (32). Additionally, published work has suggested that *A. fumigatus* efflux pumps belonging to the ATP-binding cassette (ABC) transporter family may export the toxic drugs (33–35). Also, mutations in *hapE* and in the gene encoding for the 3-hydroxy-3-methyl-glutaryl-coenzyme A (HMG-CoA) reductase, *hmg1*, have been found in multi-triazole-resistant clinical *A. fumigatus* isolates (36, 37).

Only a few studies have focused on deciphering the mechanisms of triazole resistance in *A. lentulus*. Gene replacements using *A. lentulus* and *A. fumigatus cyp51A* alleles, coupled with *in silico* VRC docking experiments employing the predicted Af*cyp51A* and Al*cyp51A* tertiary structures, supported the hypothesis that structural variations in the AlCyp51A likely play a significant role due to reduced triazole binding affinity (38–40). However, these studies were performed using a single triazole-resistant *A. lentulus* isolate, and comparisons were made with its sibling species, *A. fumigatus*. Although *A. lentulus* has been described as displaying low triazole susceptibility intrinsic to the species, recent advances in molecular taxonomy of *Aspergillus* spp. have led to the identification of *A. lentulus* isolates with heterogeneous antifungal susceptibility patterns, including isolates with reduced triazole minimal inhibitory concentrations (MICs) (11, 15, 24, 41–44). These recent findings could suggest that, even though *cyp51A* variants are responsible for the reduced triazole susceptibility in some isolates, it is not likely the only mechanism. In this study, using CRISPR-Cas9 gene editing technology and transcriptional analyzes, we attempted to determine if Cyp51, Hmg1, or the putative efflux pump AbcC is driving triazole resistance in a collection of 25 clinical *A. lentulus* isolates from the United States. Taken together, our results suggest that triazole resistance in this species is largely driven by undescribed mechanisms.

## RESULTS

### *In vitro* susceptibility testing

Using a broth microdilution assay, we tested the *in vitro* activity of AMB and three systemic triazoles currently used in clinics, that is, isavuconazole (ISA), posaconazole (PSC), and VRC, against a collection of 25 clinical *A. lentulus* isolates from the United States, and the isolates were categorized into susceptible or resistant, according to the epidemiological cutoff values (ECVs) described in the second edition of the Clinical and Laboratory Standards Institute (CLSI) document M59 (45). Although ECVs describe wild-type and non-wild-type populations, to simplify nomenclature in this manuscript, we will refer to our isolates as “susceptible” and “resistant.” All the isolates were resistant to AMB (ECV = 2 µg/mL), with MICs between eight and >32 µg/mL. Regarding VRC (ECV = 1 µg/mL), only one *A. lentulus* isolate (FH7) was considered susceptible with an MIC of 0.5 µg/mL. Most of the isolates (*n* = 22) showed an MIC of 2 µg/mL, and isolates DI19-116 and DI19-124 showed the highest levels of resistance (MICs = 8 and 16 µg/mL, respectively) (Table 1). Similar results were observed for ISA (ECV = 1 µg/mL), with three isolates showing an MIC of 1 µg/mL (FH7, DI19-126, and DI19-127), 20 isolates with an MIC of 2 µg/mL, and DI19-116 and DI19-124 showing the highest MICs (4 and 8 µg/mL, respectively). All the *A. lentulus* isolates, except isolate FH7, were considered resistant when the long-chain triazole, PSC (ECV = 0.25 µg/mL), was used, with MIC values between 0.5 and 2 µg/mL. Although clinical breakpoints have not been described for *A. fumigatus* and triazoles, and since FH7 is the isolate with the lowest VRC, ISA, and PSC MICs, all within the wild type range, we employed this isolate as our susceptible control for comparison purposes in this study. All other isolates displayed fourfold or higher VRC MICs. For the current study, we refer to these isolates as resistant.

**TABLE 1:** Amphotericin B and triazole MIC values for 25 *A*. *lentulus* clinical isolates and their Cyp51A genotype^*a*^

Isolate	MIC (µg/mL)				Cyp51A genotype
	AMB	VRC	ISA	PSC	
**DI19-114**	**16**	**2**	**2**	**1**	A9P, T11M, F29Y
**DI19-115**	**32**	**2**	**2**	**1**	A9P, T11M, F29Y
**DI19-116**	**32**	**8**	**4**	**2**	T11M, F29Y
**DI19-117**	**16**	**2**	**2**	**1**	T11M, H352Q
**DI19-118**	**16**	**2**	**2**	**2**	Identical to reference sequence
**DI19-119**	**32**	**2**	**2**	**2**	Identical to reference sequence
**DI19-120**	**16**	**2**	**2**	**1**	A9P, T11M, F29Y
**DI19-121**	**16**	**2**	**2**	**1**	Identical to reference sequence
**DI19-122**	**>32**	**2**	**2**	**0.5**	T11M, F29Y
**DI19-123**	**16**	**2**	**2**	**1**	A9P, T11M, F29Y
**DI19-124**	**16**	**16**	**8**	**1**	T11M, A12V, F29Y, E424A
**DI19-125**	**8**	**2**	**2**	**0.5**	A9P, T11M, F29Y
**DI19-126**	**>32**	**2**	1	**0.5**	T11M, F29Y
**DI19-127**	**>32**	**2**	1	**0.5**	T11M, F29Y
**DI19-128**	**16**	**2**	**2**	**0.5**	T11M, A12V, F29Y, E424A
**DI19-129**	**16**	**2**	**2**	**1**	A9P, T11M, F29Y
**DI19-130**	**8**	**2**	**2**	**1**	Identical to reference sequence
**DI19-131**	**16**	**2**	**2**	**1**	T11M, A12V, F29Y, E424A
**DI19-132**	**32**	**2**	**2**	**1**	A9P, T11M, F29Y
**DI19-133**	**>32**	**2**	**2**	**1**	T11M, A12V, F29Y, E424A
**DI16-175**	**8**	**2**	**2**	**0.5**	A9P, T11M, F29Y
**FH7**	**16**	0.5	1	0.25	V383M, V466I
**FH84**	**32**	**2**	**2**	**1**	A9P, T11M, F29Y
**FH265**	**16**	**2**	**2**	**1**	Q323R
**FH293**	**16**	**2**	**2**	**1**	A9P, T11M, F29Y

^*a*^ Values in bold are greater than the ECVs described for *A. fumigatus* (2 µg/mL for AMB, 1  µg/mL for VRC, ISA, and ITC; 0.25 µg/mL for PSC) (45).

### Sequencing of *A. lentulus cyp51A* and *cyp51B*

Like *A. fumigatus*, its sibling species, the *A. lentulus* genome contains two *cyp51*-related genes encoding 14-α-lanosterol demethylases enzymes, *cyp51A* and *cyp51B*, that share around 95% and 98% identity, respectively, with the *A. fumigatus* deduced protein sequences (39). To determine if *cyp51*-related mechanisms of triazole resistance are as important in *A. lentulus* as they are in *A. fumigatus*, we first amplified and sequenced the complete *cyp51A* gene plus 700 bp of the promoter region of every *A. lentulus* isolate from our collection. In tandem, we also sequenced the *cyp51B* gene. The list of primers utilized for both PCR amplification and sequencing can be found in Table S1. The resulting sequences were assembled and analyzed using SnapGene v4.1.9, and the predicted protein sequences were aligned using Clustal Omega (46) and compared to those of the *A. lentulus* type strain IFM 54703^T^ reference sequence as well as to other publicly available *A. lentulus* Cyp51A protein sequences (Table S2). When comparing the *A. lentulus* Cyp51A amino acid sequences, we found nine single nucleotide polymorphisms (SNPs) that generated seven different Cyp51A genotypes in our isolates (Table 1). Four isolates showed identical Cyp51A sequence to that of the type strain IFM 54703^T^ (locus TMP_alenIFM54703_5284), while the others displayed between one and four SNPs. However, none of these genomic variants appeared to reside in the putative Cyp51 triazole-binding site (38). Some of our isolates shared 100% identity with Cyp51A sequences obtained from the public database (Table S2). We noted that our most susceptible *A. lentulus* isolate, FH7, contained a unique Cyp51A genotype encoding a substitution of a valine by a methionine at position 383 and another substitution of a valine by an isoleucine at position 466. When analyzing the sequences of one of the most resistant isolates, DI19-116, we observed that the amino acid substitutions that Cyp51A harbors are not specific to this isolate. DI19-122, DI19-126, and DI19-127 have the same genotype (T11M, F29Y) and present lower MICs for all the triazoles tested, compared to DI19-116 (Table 1). Similar results were observed for the other highly pan-azole-resistant isolate, DI19-124, which showed a different combination of amino acid substitutions (T11M, A12V, F29Y, and E424A), shared with the Cyp51A protein sequence from isolates DI19-128, DI19-131, and DI19-133. Analysis of the *cyp51A* promoter region of our isolates revealed varying polymorphisms among the isolates with none residing in the putative SrbA, AtrR, CBC, or HapX binding sites, areas that have been shown to be essential for *cyp51A* regulation and activation (Data S1) (47, 48). Further, our sequencing analyses found the Cyp51B coding sequence (locus TMP_alenIFM54703_3011) to be more genetically stable, as the predicted protein sequences were identical in 24 out of our 25 isolates. DI19-119 encoded only one non-synonymous mutation that consisted of the substitution of a glutamine by a leucine at position 42 (Q42L). All the other sequences, including those retrieved from the National Center for Biotechnology Information, were identical (Table S3).

Taken together, our data suggest that triazole resistance among our *A. lentulus* isolates is likely independent of mutations in *cyp51A* and that the different genotypes observed among our collection of clinical isolates are more indicative of regular intra-species gene variability. However, as our most susceptible isolate harbored a unique *cyp51A* genotype, we hypothesized that this could be the cause of the increased susceptibility to triazoles in this isolate.

### Analysis of mRNA expression levels of *A. lentulus cyp51A* and *cyp51B*

**Fig 1:**
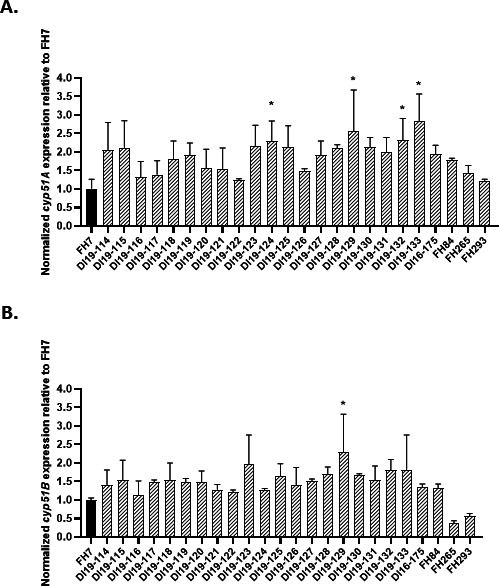
Analysis of *cyp51A* and *cyp51B* expression levels in 25 *A*. *lentulus* clinical isolates. Total RNA from each *A. lentulus* isolate was extracted after growth in glucose minimal media for 20 hours at 37°C. Expression levels of *cyp51A* (**A**) and *cyp51B* (**B**) were determined by RT-qPCR and normalized to the housekeeping gene, beta-tubulin (*tubA*). Each sample was analyzed in technical and biological triplicates. Statistics were computed by one‐way analysis of variance with Dunnett’s test for multiple comparisons in GraphPad v9.2.0 for Windows.

Studies in triazole-resistant *A. fumigatus* strains have shown that the generation of tandem repeats in the *cyp51A* promoter region (TR34, TR46, and TR53) as well as mutations in proteins of the CBC complex lead to an increased expression of the genes that encode for the target of azoles, *cyp51A and cyp51B* (33, 35, 37, 47, 49, 50). Although we did not find tandem repeat duplications in the promoter of *cyp51A* in any of our isolates, we next sought to confirm that upregulated expression of *cyp51A* or *cyp51B* was not the cause of the resistance profiles noted. To do so, we extracted total RNA from each of the 25 isolates grown for 18 hours in glucose minimal medium (GMM) at 37°C and quantified *cyp51A* and *cyp51B* transcript levels by real-time-quantitative PCR (RT-qPCR). Using our most susceptible isolate, FH7, as a control, all triazole-resistant *A. lentulus* isolates showed higher *cyp51A* expression levels, with values between 1.23- and 2.84-fold. Expression levels of *cyp51A* from four isolates (DI19-124, DI19-129, DI19-132, and DI19-133) were significantly higher than the susceptible comparator isolate (*P* ≤ 0.013) (Fig. 1A). The expression of *cyp51B* among triazole-resistant isolates ranged from 0.37- to 2.31-fold that of the susceptible comparator, FH7, with one isolate (DI19-129) exhibiting statistically significant higher expression (*P* = 0.0459) (Fig. 1B). Although our data uncover a few isolates with significantly elevated *cyp51A* or *cyp51B* expression levels when compared to the susceptible control, the overall transcriptional profile pattern does not adequately support increased target gene expression as the sole mechanism driving reduced susceptibility to triazoles among our collection.

### Azole resistance in our *A. lentulus* isolates seems *cyp51A*-independent

**Fig 2:**
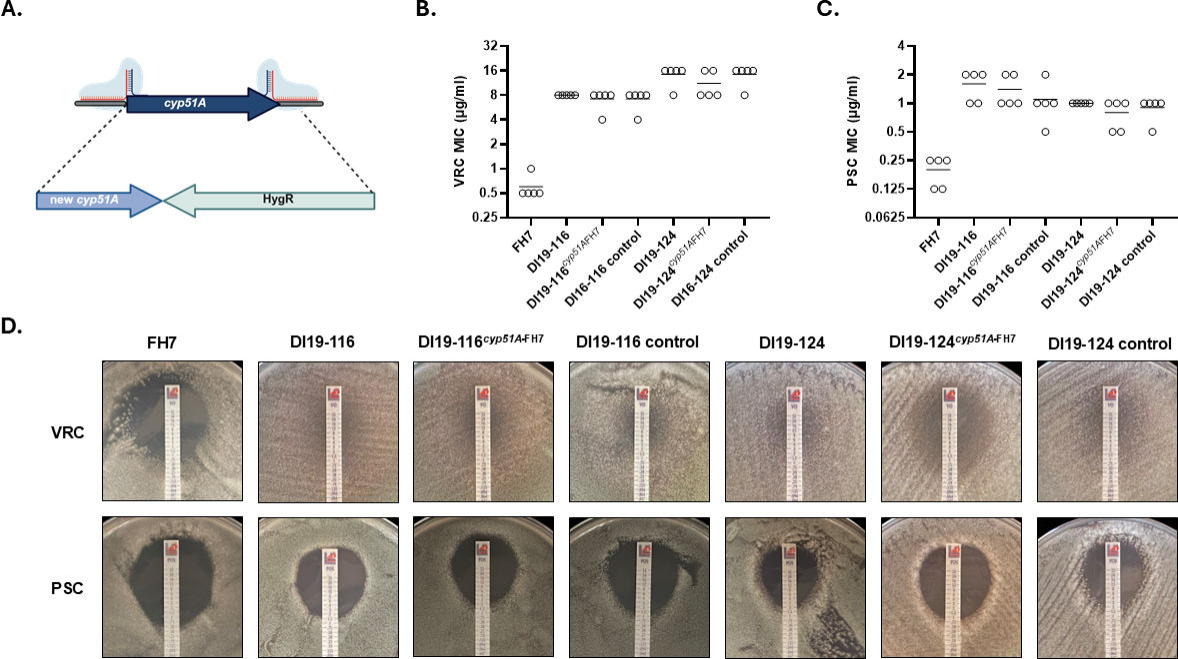
Triazole-resistant *A. lentulus* isolates expressing *cyp51A* from a susceptible isolate maintain resistance. (**A**) Schematic of *cyp51A* allele replacement. Two suitable PAM sites near the 5′- and 3′-ends of the *cyp51A* of interest were targeted with a repair template carrying the new *cyp51A* allele upstream of a hygromycin resistance cassette. (**B and C**) The complete *cyp51A* ORFs from the isolates with highest triazole MICs (DI19-116 and DI19-124) were replaced with the *cyp51A* allele from a susceptible isolate (FH7), following the strategy described in Fig. 2A. VRC (**B**) and PSC (**C**) MICs were determined by broth microdilution, following the recommendations of the CLSI M38 document (52). Circles represent biological replicates, and horizontal lines indicate mean values. (**D**) *In vitro* susceptibility of parental and mutant *A. lentulus* strains using antifungal test strips on RPMI agar. Pictures were taken after 48 hours of incubation at 35°C and are representative of biological triplicates. Note that the resistant phenotype of DI19-116 and DI19-124 is not altered after *cyp51A* allele replacement.

Our *cyp51A* sequencing data uncovered that our susceptible isolate, FH7, harbored a unique genotype not observed in other isolates from our collection. To genetically test if this unique *cyp51A* genotype underpins reduced triazole susceptibility, we next replaced the complete *cyp51A* ORF of the pan-azole-resistant isolates (DI19-116 and DI19-124) with the FH7 *cyp51A* allele. A targeted allele-swap gene replacement was obtained using a CRISPR-Cas9 technique, previously established for gene targeting in *A. fumigatus* in our laboratory (51) (Fig. 2A), and the resulting strains were named DI19-116*^cyp51A^*^FH7^ and DI19-124*^cyp51A^*^FH7^. These new strains express the integrated *cyp51A* alleles from the endogenous promoter. Manipulation control strains containing a hygromycin resistance cassette incorporated downstream of the endogenous *cyp51A* allele were also built in each isolate background to ensure that the mutation did not cause a change in antifungal susceptibility. These control strains were named “DI19-116 control” and “DI19-124 control.”

Antifungal susceptibility testing performed by a broth microdilution assay (52) showed that DI19-116 and DI19-124 have VRC MICs of 8 and 16 µg/mL, respectively. These values are up to 32-fold higher than those observed for isolate FH7 (Table 1; Fig. 2). As shown in Fig. 2B, when *cyp51A* from these isolates was replaced with the FH7 *cyp51A* allele, we did not observe a change in MIC, with all the replicate values falling within one dilution. Similar results were observed when the long-chain azole, PSC, was tested. The parental resistant strain DI19-116 showed MICs within the range of 1–2 µg/mL, and replacing *cyp51A* with the FH7 allele showed the same trend. Similarly, when analyzing PSC susceptibility in the DI19-124 isogenic strains, we observed MICs of 1 and 0.5–1 µg/mL for parental and DI19-124*^cyp51A^*^FH7^ mutant, respectively. Control strains had MIC values for both triazoles similar to their respective parental isolates (Fig. 2B and C).

When *in vitro* susceptibility to VRC and PSC was determined by antifungal drug diffusion strips, we observed similar resistance patterns (Fig. 2D, upper panel). Unlike isolate FH7, which showed a VRC MIC of 0.25 µg/mL, DI19-116 and DI19-124 did not generate a clear growth inhibition zone around the VRC drug strip, and consequently, the MIC was >32 µg/mL. All the mutants generated in these genetic backgrounds had the same VRC MIC, ensuring the elevated resistance phenotype (Fig. 2D, upper panel). When testing PSC, isolate FH7 showed an MIC of 0.5 µg/mL, while DI19-116 and DI19-116*^cyp51A^*^FH7^ had MICs of 1.5 and 2 µg/mL, respectively (Fig. 2D, lower panel). Isolate DI19-124 and DI19-124*^cyp51A^*^FH7^ showed MICs of 2 and 1 µg/mL, respectively. Control strains’ MICs for both triazoles were the same as obtained for their parental isolates (Fig. 2D). These results confirmed that *cyp51A* allele replacements between triazole-susceptible and triazole-resistant *A. lentulus* strains do not alter VRC or PSC MICs, suggesting that antifungal resistance in this cryptic species is driven by mechanisms independent of *cyp51A*.

**Fig 3:**
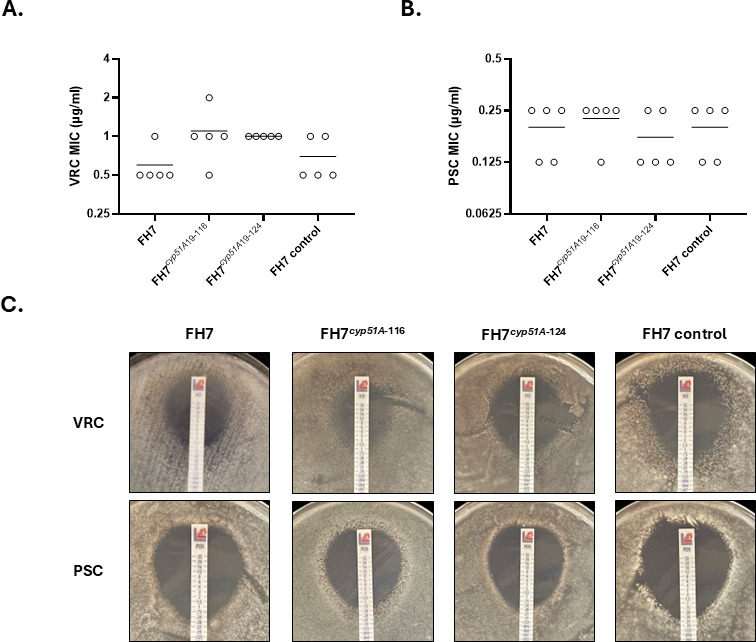
A triazole-susceptible *A. lentulus* isolate expressing *cyp51A* alleles from resistant isolates maintains susceptibility. The complete *cyp51A* coding sequence from isolate FH7 (triazole-susceptible) was replaced by the *cyp51A* allele of DI19-116 or DI19-124 (triazole-resistant isolates), following the strategy described in Fig. 2A. VRC (**A**) and PSC (**B**) MICs were determined by broth microdilution, following the recommendations of the CLSI M38 document (52). Circles represent biological replicates and horizontal lines indicate mean values. (**C**) *In vitro* susceptibility of parental and mutant *A. lentulus* strains using antifungal test strips. Pictures were taken after 48 hours of incubation at 35°C and are representative of biological triplicates. Note that the triazole-susceptible phenotype of FH7 does not change after *cyp51A* allele replacement.

To further confirm that *cyp51A* is not responsible for triazole resistance in DI19-116 and DI19-124, we expressed their *cyp51A* alleles in our susceptible isolate FH7 genetic background, following the same CRISPR-Cas9 gene editing strategy shown in Fig. 2A. Repair templates containing the complete *cyp51A* allele from either DI19-116, DI19-124, or FH7 (for the control strain), fused to a hygromycin resistance cassette, were incorporated into the *cyp51A* native locus of isolate FH7. These strains were named FH7*^cyp51A^*^19-116^ and FH7*^cyp51A^*^19-124^. MIC broth microdilution analyses of five biological replicates for parental (FH7) and mutant strains revealed MICs ranging from 0.5 to 1 µg/mL for FH7 and FH7 control strains, whereas the mutant FH7*^cyp51A^*^19-116^ MIC ranged from 0.5 to 2 µg/mL. Expression of DI19-124 *cyp51A* in FH7 resulted in VRC MIC of 1 µg/mL (Fig. 3A). PSC MICs were identical between parental and control FH7 strains and similar to the mutants’ MICs, with only one twofold dilution change for some of the replicates (Fig. 3B). When antifungal susceptibility was determined using a drug strip diffusion assay in RPMI agar, we observed that the area of growth inhibition by the FH7*^cyp51A^*^19-116^ mutant was slightly smaller than that observed with the parental susceptible strain, although the VRC MIC was only twofold higher (MIC = 2 µg/mL for FH7*^cyp51A^*^19-116^ vs MIC = 1 µg/mL for FH7). However, when *cyp51A* from DI19-124 was expressed in FH7, the resulting strain showed a VRC MIC of 0.5 µg/mL (Fig. 3C, upper panel). When PSC was tested, all the strains showed similar results, with FH7 and FH7*^cyp51A^*^19-124^ showing an MIC of 0.5 µg/mL and FH7*^cyp51A^*^19-116^ showing an MIC of 0.75 µg/mL (Fig. 3C, lower panel). Therefore, our allele swap experiments suggest that *cyp51A* is not responsible for the triazole resistance phenotype observed in our *A. lentulus* isolates DI19-116 and DI19-124.

### Analysis of mRNA expression of the *A. lentulus* ABC transporter, *abcC*

**Fig 4:**
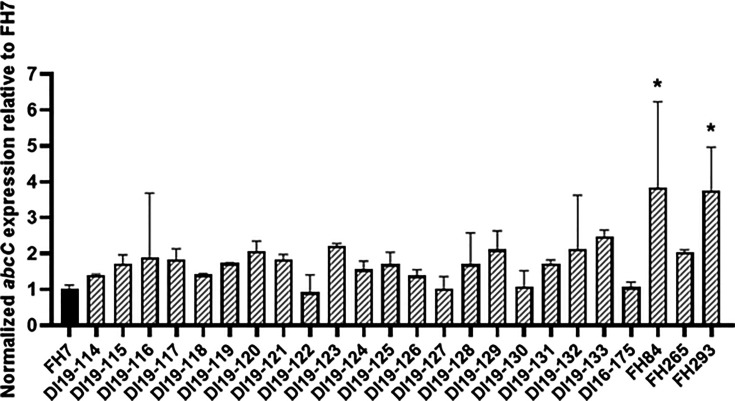
Analysis of the efflux pump, *abcC,* expression levels in 25 *A*. *lentulus* clinical isolates. Total RNA from each *A. lentulus* isolate was extracted in biological triplicates, and expression levels were determined by RT-qPCR as explained in Fig. 1 and Materials and Methods. Statistics were computed by one‐way analysis of variance with Dunnett’s test for multiple comparisons in GraphPad v9.2.0 for Windows.

Studies in *A. fumigatus* have shown that, in addition to Cyp51-mediated triazole resistance mechanisms, increased expression of efflux pumps has also been associated with low susceptibility to triazoles (33, 35, 53). Since our allele swap experiments uncovered no correlation between *cyp51A* and *cyp51B* genotype or expression and triazole resistance pattern in our collection of *A. lentulus* isolates, we decided to next explore the potential role of the putative *Aspergillus* triazole efflux pump AbcC/Cdr1B. We first identified a predicted ortholog of *A. fumigatus* AbcC/Cdr1B in *A. lentulus* through BLASTP search of the *A. lentulus* reference strain IFM 54703^T^ genome using the *A. fumigatus* Af293 AbcC protein sequence (Afu1g14330) as a query. This search revealed one homolog with 98% of identity, encoded by the locus TMP_alenIFM54703_8032, that we subsequently named AbcC (54). When comparing gene expression levels of the resistant strains versus FH7 by RT-qPCR, we observed that *abcC* expression ranged from 0.92- to 3.84-fold that of the susceptible isolate, and only *abcC* expression in FH84 and FH293 was statistically higher (Fig. 4). Together, these data suggest that expression levels of the AbcC efflux pump alone are likely not a major driver of the intrinsic low susceptibility to triazoles observed in our *A. lentulus* collection.

### Sequencing of *hmg1* reveals mutations in three *A. lentulus* triazole-resistant isolates

Recent studies in *A. fumigatus* have investigated novel molecular mechanisms that confer resistance to triazoles that are independent of Cyp51 and drug efflux. Multiple studies using resistant clinical isolates have revealed that mutations in the HMG-CoA reductase (Hmg1) sterol sensing domain generate reduced susceptibility to triazoles, likely through altering sterol intermediate-induced negative feedback of the Hmg1 protein (36, 55). To evaluate the role of *hmg1* in our collection of *A. lentulus* clinical isolates, we first identified a predicted ortholog of Hmg1 in this species, using the *A. fumigatus* Af293 Hmg1 (Afu2g03700) protein sequence as a query in a BLASTP search in FungiDB (54). Results revealed that the locus TMP_alenIFM54703_4593, which encodes for a 3-hydroxy-3-methylglutaryl-coenzyme A reductase, shares 96% identity with *A. fumigatus* Hmg1. To identify possible resistance-associated mutations in *hmg1*, the complete *hmg1* ORF of each isolate was PCR-amplified and Sanger-sequenced using primer sets described in Table S1. Sequencing results revealed that all isolates except three shared identical *hmg1* genotypes. The three isolates harboring SNPs compared to the FH7 control were DI19-120, with a mutation at position 659 resulting in the substitution of a serine for a proline; DI19-121, encoding a glutamic acid-to-lysine switch at position 672; and DI19-116, encoding a phenylalanine-to-leucine at position 420. Importantly, the F420L substitution found in isolate DI19-116 resides within the sterol sensing domain of Hmg1. We therefore hypothesized that the F420L mutation could play a role in the resistance phenotype observed in *A. lentulus* isolate DI19-116.

**Fig 5:**
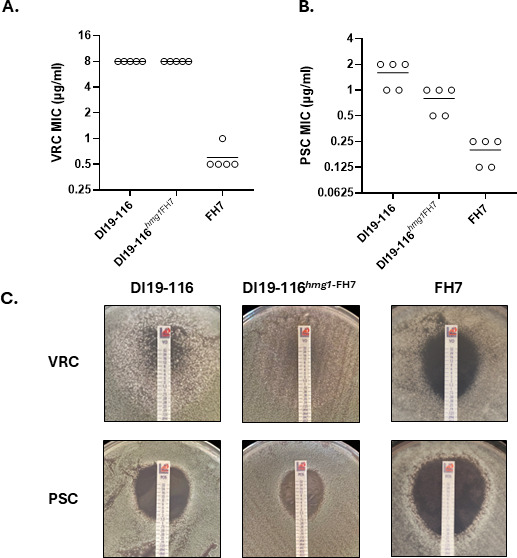
*hmg1* is not responsible for the triazole resistance phenotype in *A. lentulus* isolate DI19-116. The complete *hmg1* coding sequence from the triazole-resistant isolate, DI19-116, was replaced with the *hmg1* allele from the susceptible isolate, FH7, following the strategy described in Fig. 2A. VRC (**A**) and PSC (**B**) MICs were determined by broth microdilution, following the recommendations of the CLSI M38 document (52). Circles represent biological replicates, and horizontal lines indicate mean values. (**C**) *In vitro* susceptibility of parental and mutant *A. lentulus* strains using antifungal test strips on RPMI agar. Pictures were taken after 48 hours of incubation at 35°C and are representative of biological triplicates. Note that *hmg1* allele replacement does not affect the resistance phenotype observed in DI19-116.

To test this hypothesis, we replaced the *hmg1* native locus from DI19-116 with the complete *hmg1* ORF from our susceptible isolate, FH7, using our CRISPR-Cas9 gene editing technique (51). Antifungal susceptibility testing was then performed by broth microdilution for VRC and PSC with FH7 (susceptible isolate), DI19-116 (parental, triazole-resistant strain), and the mutant strain DI19-116*^hmg^*^1FH7^. Figure 5A shows that genetic manipulation of the *hmg1* locus did not alter VRC MIC as both parental and mutant strains displayed an MIC of 8 µg/mL, which is 16-fold higher than the observed for FH7, with an MIC of 0.5 µg/mL. When evaluating PSC susceptibility, the parental strain MIC values ranged from 1 to 2 µg/mL, while FH7 PSC MICs ranged from 0.125 to 0.25 µg/m. The mutant DI19-116*^hmg^*^1FH7^ MICs for this drug ranged from 0.5 to 1 µg/mL (Fig. 5B). When *in vitro* susceptibility testing was performed using diffusion drug strips, both DI19-116 and DI19-116*^hmg^*^1FH7^ showed VRC resistance phenotypes, with fungal growth covering the entire area (MIC ≥ 32 µg/mL) (Fig. 5C, upper panel), corresponding to at least 64-fold higher MIC values than those observed for isolate FH7. PSC MICs for the DI19-116 isogenic strains and FH7 were 1.5 and 1 µg/m, for DI19-116 and DI19-116*^hmg^*^1FH7^, respectively, whereas FH7 PSC MIC was 0.38 µg/mL (Fig. 5C, lower panel). These results suggest that the F420L mutation, residing within the sterol sensing domain of the DI19-116 clinical isolate, was not responsible for the elevated triazole MICs.

## DISCUSSION

*A. lentulus* is an emerging fungal pathogenic cryptic species with indistinguishable morphology from *A. fumigatus* that generally presents low susceptibility to triazoles, caspofungin, and AMB (6, 14). Studies from recent years suggest that cryptic species are becoming clinically relevant, representing the cause of around 11-30% of IA cases in both neutropenic and non-neutropenic patients (11, 12, 43, 56, 57). However, these studies probably represent just a fraction of the total cases of IA that happen worldwide, mainly because accurate species-level identification by molecular methods is not always accessible.

*A. fumigatus* continues to be the leading causative agent of IA, and due to its higher prevalence, the molecular mechanisms responsible for antifungal resistance are actively being investigated in this species. For example, it is well-known that certain amino acid substitutions, as well as repetitions in the promoter of one of the genes encoding for the target of the triazole drugs, *cyp51A*, are common and responsible for resistance to this class of antifungals (reviewed in [32]). Our results presented here support the hypothesis that known resistance mechanisms are not the major drivers of increased triazole MICs in our clinical isolate library. Sequencing of *cyp51A* and its promoter region revealed intra-species genetic variations in our *A. lentulus* collection, observed in previously sequenced isolates (39, 40, 44). Analysis of the promoter region of *cyp51A* also revealed genetic variations between isolates (Data S1), but tandem repetitions or alterations in the AtrR, SrbA, CBC, or HapX binding sites were not observed. This is in agreement with dos Santos et al. (44), where whole genome sequencing and phenotypic characterization were performed in clinical *Aspergillus* section *Fumigati* isolates, and, although genetic variation was found in genes important in antifungal resistance and virulence that may be associated with phenotypic heterogeneity, they did not find genetic alterations in areas important for Cyp51A regulation (44). CRISPR-Cas9-driven allele swaps of *cyp51A* between our most susceptible (FH7) and most resistant (DI19-116 and DI19-124) *A. lentulus* isolates show that triazole resistance is independent of *cyp51A*. A previous study showed that heterologous expression of a *cyp51A* allele from a triazole-resistant *A. lentulus* in a drug-susceptible *A. fumigatus akuB*^KU80^ genetic background caused the susceptible isolate to become resistant to triazoles (39). mRNA expression analyses by RT-qPCR showed similar *cyp51A* expression between the parental *A. fumigatus* isolate and the mutant strains containing the *A. lentulus cyp51A* ORF (39). However, expression of *Alcyp51A* was driven under the *A. fumigatus* promoter and 3′UTR, and little is known about the post-transcriptional or translational regulation of *cyp51A* in this heterologous expression scenario. Moreover, using *in silico* drug modeling and VRC docking with both *A. fumigatus* and *A. lentulus* Cyp51A predicted 3D structures, the authors concluded that triazole resistance might be due to a low affinity and unstable Cyp51A-VRC binding underpinned by amino acid sequence differences between the two species in areas close to the catalytic core (38). However, this conclusion does not fully explain why some *A. lentulus* isolates have been characterized as triazole-susceptible (like FH7 in this study) as they share the same sequence in the VRC-binding area as triazole-resistant isolates. More recently, another study described the expression of a triazole-susceptible *A. fumigatus cyp51A* allele in a triazole-resistant *A. lentulus* isolate. The resulting mutants displayed significant reductions of itraconazole and VRC MICs, reaching wild type levels (40). These studies again suggest that triazole resistance is being driven by *cyp51A* in the *A. lentulus* isolates studied. Both mentioned studies employ one single *A. fumigatus* or *A. lentulus* isolate as parental background for the *cyp51A* heterologous expression experiments. The advances in fungal genetic engineering and the development of CRISPR-Cas9 gene-editing techniques now allow for rapid generation of mutant strains. Therefore, it would be interesting to repeat these assays in different genetic backgrounds to account for any strain-specific events. The observations from the present work and previous studies may also suggest that different *A. lentulus* isolates harbor different or multiple triazole resistance mechanisms, as observed in *A. fumigatus*. For example, increased expression of the gene encoding for the ABC efflux pump, *abcC* (also known as *cdr1B* or *abcG1*), has also been associated with antifungal resistance in *A. fumigatus*, and null mutants in this efflux pump show significant reduction in VRC, PSC, and itraconazole MICs, compared to their parental strains (33, 35, 53). From our isolates, only two presented significantly increased *abcC* expression levels, compared to the most susceptible, FH7 (Fig. 5A). However, the expression of the efflux pump was lower than that reported to drive resistance in other studies (33). Therefore, we cannot conclude that export of triazoles by AbcC is fully responsible for the resistance phenotype in these cases. In addition, two recent studies described mutations in the gene encoding the HMG-CoA reductase enzyme, *hmg1*, to be responsible for *cyp51A*-independent triazole resistance in *A. fumigatus* (36, 55). Three of our resistant *A. lentulus* isolates harbor SNPs in *hmg1*, and for one of them (DI19-116), the sterol-sensing domain was affected, possibly causing a dysregulation of the ergosterol biosynthesis pathway, as shown in *A. fumigatus* (36, 55). However, the expression of *hmg1* from our most susceptible isolate (FH7) in DI19-116 did not cause an alteration in antifungal susceptibility; therefore, concluding that the identified mutation does not affect enzyme activity in this particular isolate and that resistance to triazoles is driven by other, unknown mechanisms.

A limitation of this study is that gene expression analyzes were determined under basal growth conditions and not in the presence of triazole stress. Including such studies could further uncover differential antifungal responses that underpin resistance in *A. lentulus*. However, we have initially deciphered basal mRNA expression levels alone as our current collection only contains one “susceptible” control isolate. Therefore, comparisons would likely not be representative of *A. lentulus* as a species. Further investigations will be needed to fully decipher the complexity of triazole stress response and resistance in the cryptic species *A. lentulus*. The access to whole genome and transcriptome sequencing data, as well as the rapid development of genetic engineering tools adapted to cryptic species, will provide additional needed data that will expedite the study of *Aspergillus* biology in different species and will assist in identification of novel mechanisms of antifungal resistance.

## MATERIALS AND METHODS

### Strains and culture conditions

Twenty-five *A. lentulus* clinical isolates from the United States (kindly provided by Dr. N. Wiederhold and Dr. K. Marr) were used in this study (Table 1). All the isolates were grown in GMM (6 g NaNO_3_, 0.52 g KCl, 0.52 g MgSO_4_ × 7H_2_O, 1.52 g KH_2_PO_4_, 1 mL trace elements [2.2 g ZnSO_4_ × 7H_2_O, 1.1 g H_3_BO_3_, 0.5 g MnCl_2_ × 4H_2_O, 0.5 g FeSO_4_ × 7H_2_O, 0.16 g CoCl_2_ × 5H_2_O, 0.16 g CuSO_4_ × 5H_2_O, (NH_4_)_6_Mo_7_O_24_ × 4H_2_O, 5 g Na_4_EDTA in 100 mL distilled H_2_O], 10 g glucose, 15 g agar, pH 6.5, in 1 L distilled H_2_O) at 37°C for 7–10 days, or until enough conidia were generated. Conidial stocks were preserved in 30% glycerol at −80°C. Conidia were harvested by flooding the plates with dH_2_O, filtering through a Miracloth, and washing twice with dH_2_O. Appropriate inocula concentrations were adjusted by counting with a hemocytometer.

### *In vitro* susceptibility testing

*In vitro* activity of AMB, ISA, PSC, and VRC was determined after 48 hours in RPMI at 35°C, using the broth microdilution susceptibility testing protocol described in the CLSI M38 document (52). The MICs were read after 48 hours of growth at 35°C and determined by the concentration of the first well presenting a 100% of growth inhibition. *Candida krusei* ATCC 6258 was used as quality control strain to ensure proper activity of each antifungal drug. In addition, MICs were determined using antifungal test strips (Liofilchem), following the manufacturer’s instructions. Briefly, conidia suspensions were prepared in sterile dH_2_O and adjusted to a concentration of 10^6^ conidia/mL. RPMI agar plates were inoculated by dipping a sterile swab into the cell suspension and streaking it across the surface of the agar in three directions. The plates were allowed to dry completely before applying the VRC, ISA, or PSC test strips. The plates were incubated at 35°C for 48 hours. The MIC was recorded as the drug concentration at the point where dense colonial growth intersected the strip. Each experiment was repeated at least three times on different days.

The isolates were categorized into susceptible and resistant, considering the ECVs published in the second edition of the document M59 from the CLSI (45). In this sense, the strains with an MIC for AMB lower than or equal to 2 µg/mL or VRC or ISA lower than or equal to 1 µg/mL were considered susceptible. The strains showing higher MICs were considered resistant, suggesting that they possess an intrinsic or acquired drug resistance mechanism. In the case of PSC, the CLSI has not approved interpretive endpoints for this drug and *A. fumigatus* due to an overlap between MICs for presumptive wild type and mutant isolates. However, a multicenter study of PSC MIC distributions for *A. fumigatus* species complex proposes an ECV of 0.25 µg/mL for this drug/species combination (58). Therefore, we have considered resistant strains to be those that had a PSC MIC higher than 0.25 µg/mL.

### *Cyp51A*, *cyp51B*, and *hmg1* sequencing

The entire *cyp51A* allele ranging from −700 bp to the predicted STOP codon and the complete *cyp51B* and *hmg1* coding sequences were amplified from genomic DNA with Kappa HiFi DNA polymerase (Roche), using the primers shown in Table S1. The PCR products were then purified using the GeneJet PCR Purification Kit (Thermo Scientific), according to the manufacturer’s instructions and Sanger sequenced in both directions. Protein sequences were deduced using SnapGene 4.1.9 for Windows, and aligned in Clustal Omega (46).

### mRNA expression analysis

Gene expression profiles of *cyp51A* (TMP_alenIFM54703_5284) and *cyp51B* (TMP_alenIFM54703_3011) and the putative ABC transporter ortholog of the *A. fumigatus* AbcC (TMP_alenIFM54703_8032) were determined by RT-qPCR. Total RNA was extracted from each of the 25 *A*. *lentulus* isolates, grown in GMM supplemented with 0.5% yeast extract (GMM-YE) for 18 hours at 37°C and constant agitation (250 rpm), as previously described (59). Quality and quantity of RNA was determined spectrophotometrically by Nanodrop. Five micrograms of total RNA were digested with Turbo DNAse (Invitrogen) to remove any genomic DNA contaminant, and 1 µg of RNA was then used for cDNA synthesis using the ProtoScript II Kit (New England Biolabs). Finally, cDNA was treated with RNAase H (New England Biolabs) for 20 minutes at 37°C. RT-qPCR was performed in a CFX Connect Real‐Time System (BioRad) using the SYBR Green Master Mix (Bio‐Rad) and gene-specific primers (Table S1). Relative expression of *cyp51A*, *cyp51B*, and *abcC* was normalized to that of beta-tubulin (locus TMP_alenIFM54703_8364) (*tubA*). Changes in gene expression between isolates were calculated using the 2^ΔΔCt^ method (60). Each experiment was performed independently three times with three technical replicates.

### Genetic manipulations

The *cyp51A* coding sequences from *A. lentulus* isolates FH7, DI19-116, and DI-19-124 were PCR-amplified and sub-cloned upstream of a hygromycin resistance (hygR) cassette in the previously generated pCyp51A-HygR plasmid (36). Repair templates containing the *cyp51A-hygR* cassette were PCR-amplified with primers incorporating approximately 40 bp of microhomology to the 5′- and 3′-ends of the target sequences. For allele replacements, we adapted our *A. fumigatus* CRISPR-Cas9 gene targeting system (51) to *A. lentulus*. Briefly, two protospacer adjacent motif (PAM) sites were chosen close to the *cyp51A* start and stop codons, and the protospacer consisted of the 20 nucleotides immediately upstream of the PAM site. The guide RNAs (gRNAs) were assembled *in vitro* by mixing equal amounts of transactivating CRISPR RNA, the corresponding CRISPR RNA, and duplexing buffer and boiling at 95°C for 5 minutes. The ribonucleoprotein complexes required for gene targeting were built by combining the gRNAs with the Cas9 enzyme and incubating at room temperature for 15 minutes, as previously described (51).

For *hmg1* allele replacement, the complete *hmg1* coding sequence of *A. lentulus* isolate FH7 was PCR-amplified and sub-cloned into the plasmid pAGRP, which contains a phleomycin resistance cassette, using the restriction sites *XbaI* and *NotI* (61). The repair template was PCR-amplified to again incorporate approximately 40 bp of microhomology to the 5′- and 3′-ends of the target site. PAM site selection and RNP generation were carried out as described above.

Protoplast generation and transformations were carried out as previously described (62). Approximately 1.2 × 10^8^ conidia were grown for 18 hours in 100 mL of yeast glucose medium at 32°C and 250 rpm. After this time, the hyphal material was filtered and washed with abundant dH_2_O and incubated for 2 hours at 32°C and 75 rpm with an enzymatic solution consisting of β-glucanases and poly-galacturonases (5% wt/vol) (VinoTaste, Novozymes, Bagsværd, Denmark) in osmotic medium solution (1.2 M MgSO_4_ × 7H_2_O, 10 mM sodium phosphate buffer). The release of protoplasts was monitored every hour. When ready, the protoplasts were recovered as an intermediate cotton-like layer after centrifugation with trapping buffer (0.6 M sorbitol, 100 mM Tris-HCl, pH 7.0) and washed once with STC (1.2 M sorbitol, 7.55 mM CaCl_2_⋅H_2_O, 10 mM Tris-HCl, pH 7.5). Then, 0.2 mL containing 10^6^ protoplasts were incubated for 50 minutes on ice with the RNP complexes, 2 µg of DNA repair templates, and 25 µL of 60% PEG-CaCl_2_ solution (60% [wt/vol] PEG 3350, 50 mM CaCl_2_⋅H_2_O, 450 mM Tris-HCl, pH 7.5). After this time, 1.25 mL of 60% PEG-CaCl_2_ was added and incubated at room temperature for 20 additional minutes. Then, the transformation mixtures were plated on Sorbitol Minimal Medium (SMM) plates and the protoplasts were allowed to recover overnight at room temperature. For selection of transformant colonies, the plates were overlayed with top agar (SMM containing 7.5 g/L of agar) plus 150 µg/mL of Hygromycin B Gold (Invivogen) or 125 µg/mL of phleomycin (Invivogen). The plates containing hygromycin were placed at 37°C, while the ones containing phleomycin were incubated overnight at room temperature and then transferred to 30°C, until colonies were observed. Potential transformants were screened by multiple diagnostic PCR reactions using primers with complementarity to sites both internal and external to the repair template, as well as by Sanger sequencing of the target regions.

## SUPPLEMENTAL MATERIAL

Data S1 aac.00690-25-s0001.pdf Nucleotide sequence alignment of cyp51A promoter sequences of 25 *A. lentulus* isolates.

Supplemental tables aac.00690-25-s0002.xlsx Tables S1 to S3.

ASM does not own the copyrights to Supplemental Material that may be linked to, or accessed through, an article. The authors have granted ASM a non-exclusive, world-wide license to publish the Supplemental Material files. Please contact the corresponding author directly for reuse.
